# Single-Center Overview of Pediatric Monogenic Autoinflammatory Diseases in the Past Decade: A Summary and Beyond

**DOI:** 10.3389/fimmu.2020.565099

**Published:** 2020-09-17

**Authors:** Wei Wang, Zhongxun Yu, Lijuan Gou, Linqing Zhong, Ji Li, Mingsheng Ma, Changyan Wang, Yu Zhou, Ying Ru, Zhixing Sun, Qijiao Wei, Yanqing Dong, Hongmei Song

**Affiliations:** Department of Pediatrics, Peking Union Medical College Hospital, Chinese Academy of Medical Sciences, Beijing, China

**Keywords:** autoinflammatory diseases, innate immunity, genetic sequencing, pediatric immunology, clinical rheumatology

## Abstract

**Objective:** Monogenic autoinflammatory diseases (AIDs) are inborn disorders caused by innate immunity dysregulation and characterized by robust autoinflammation. We aimed to present the phenotypes and genotypes of Chinese pediatric monogenic AID patients.

**Methods:** A total of 288 pediatric patients clinically suspected to have monogenic AIDs at the Department of Pediatrics of Peking Union Medical College Hospital between November 2008 and May 2019 were genotyped by Sanger sequencing, and/or gene panel sequencing and/or whole exome sequencing. Final definite diagnoses were made when the phenotypes and genotypes were mutually verified.

**Results:** Of the 288 patients, 79 (27.4%) were diagnosed with 18 kinds of monogenic AIDs, including 33 patients with inflammasomopathies, 38 patients with non-inflammasome related conditions, and eight patients with type 1 interferonopathies. Main clinical features were skin disorders (76%), musculoskeletal problems (66%), fever (62%), growth retardation (33%), gastrointestinal tract abnormalities (25%), central nervous system abnormalities (15%), eye disorders (16%), ear problems (9%), and cardiopulmonary disorders (8%). The causative genes were *ACP5, ADA2, ADAR1, IFIH1, LPIN2, MEFV, MVK, NLRC4, NLRP3, NLRP12, NOD2, PLCG2, PSMB8, PSTPIP1, TMEM173, TNFAIP3, TNFRSF1A*, and *TREX1*.

**Conclusions:** The present study summarized both clinical and genetic characteristics of 18 kinds of monogenic AIDs found in the largest pediatric AID center over the past decade, with fever, skin problems, and musculoskeletal system disorders being the most prevalent clinical features. Many of the mutations were newly discovered. This is by far the first and largest monogenic AID report in Chinese pediatric population and also a catalog of the phenotypic and genotypic features among these patients.

## Introduction

Autoinflammatory diseases (AIDs) are a group of disorders characterized by remarkable inflammation without high-titer autoantibodies or antigen-specific T cells (1). Most of AIDs harbor a single gene defect, hence called monogenic AIDs, while other disorders like PFAPA are polygenic AIDs (2). Since the identification of *MEFV*, a gene causing Familial Mediterranean Fever (FMF) once mutated (3), many monogenic AIDs have been identified over the past two decades assisted by the development of genetic sequencing technologies. According to the recent 2017 International Union of Immunological Societies (IUIS) classification, there are 36 monogenic AIDs across three branches, defects affecting the inflammasomes (inflammasomopathies), non-inflammasome related conditions, and type 1 interferonopathies (4).

An inflammasome is a multiprotein pro-inflammatory complex consisting of a sensor protein like NOD-like receptor (NLR) containing protein or pyrin, an adapter protein such as apoptosis-associated speck-like protein (ASC) and an effector protein such as caspase-1 (5). The sensors recognize pathogen associated molecular patterns (PAMPs) or danger associated molecular patterns (DAMPs) to initiate the assembly and activation of inflammasomes, thereby cleaving interleukin-1 (IL-1) and freeing IL-1β and IL-18 to cause further inflammation (6). The inflammasomopathies then denote a group of mechanistically similar diseases, resulting in inappropriate inflammasome activation (7). Non-inflammasome related conditions are a group of heterogeneous disorders such as Blau syndrome, pyogenic arthritis, pyoderma gangrenosum, and acne (PAPA) syndrome, and ADA2 deficiency (8). Each one arises from different genetic mutations. As for type 1 interferonopathies, they are uniquely characterized by an inappropriate overproduction of type 1 interferon (IFN-I) (9). Aicardi-Goutieres syndrome (AGS) and proteasome-associated autoinflammatory syndrome (PRAAS), such as chronic atypical neutrophilic dermatosis with lipodystrophy and elevated temperature (CANDLE), are two typical syndromes that belong to this category. However, this field is still rapidly advancing and newly discovered diseases, such as DNaseII deficiency (10), RIPK1 deficiency (11), are periodically added to the list.

Arriving at a final definite diagnosis of monogenic AID can be very difficult because of the multifaceted nature of the disease. A strong clinical clue plus solid genetic findings can help reach a correct diagnosis. In this report, we aim to provide an overview of monogenic AIDs diagnosed by a tertiary Chinese pediatric rheumatology disease center based in Beijing over the past decade.

## Methods

### Patient Selection

**Figure 1:**
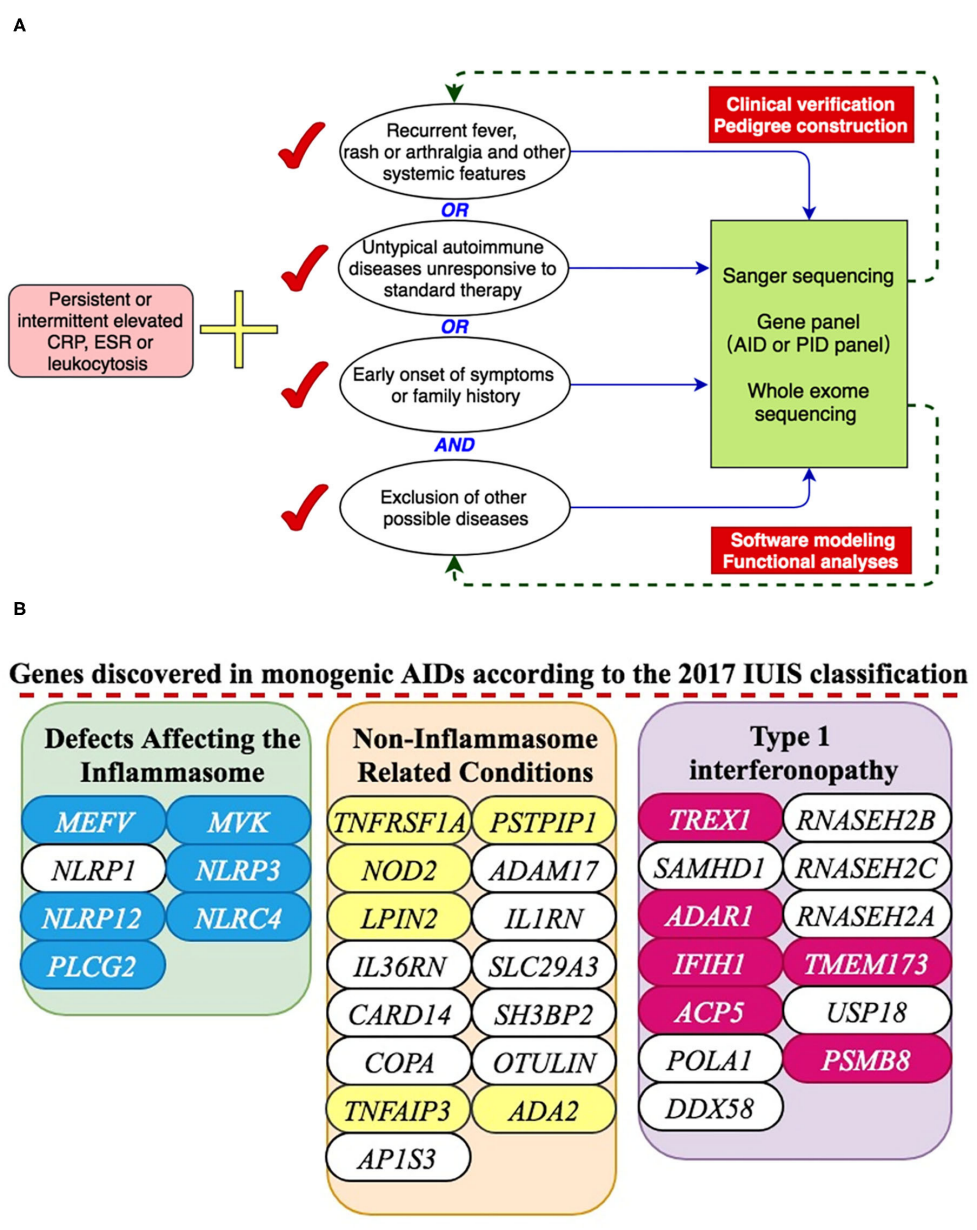
A presentation of monogenic AID gene discovery. **(A)** Inclusion criteria for suspected AID patients and working flow of genetic analyses. **(B)** 18 genes were discovered in our center according to the 2017 IUIS classification.

A total of 288 pediatric patients (age 18 or less) suspected of having monogenic AIDs were included in this study at the Peking Union Medical Hospital between November 2008 and May 2019. They all had persistently or intermittently elevated inflammation indices with no evidence of malignancy or infection. In addition to unexplained elevated inflammation markers, AID diagnosis may be considered if a patient met one or more of the following three criteria as depicted in Figure 1A: (1) presenting with recurrent fever, rash and/or arthralgia and other systemic features; (2) diagnosed with autoimmune diseases previously but unresponsive to standard therapies; (3) had family history or had early onset symptoms. All these patients and legal guardians gave informed consent for genetic sequencing. The clinical information of all these patients was obtained from paper and electronic medical records. There was no consanguinity in these children. The Institutional Review Board of the Peking Union Medical College Hospital approved this study.

### Genetic Analyses

We ordered genetic tests primarily based on the clinical pictures. For cases suspected of FMF, Blau syndrome, cryopyrin associated periodic syndrome (CAPS) or other very typical cases, the genomic DNA from peripheral blood samples of those suspected patients were genotyped for specific candidate genes such as *MEFV, NOD2, NLRP3*, or other specific AID genes using Sanger sequencing. For cases with overlapping clinical manifestations or previous negative Sanger sequencing, a gene panel including 347 primary immunodeficiency disease gene (PID panel) or Whole exome sequencing (WES) method would be recommended and utilized given patient family approval. Variants found in patients would be stringently analyzed with different information tools such as the Exome Aggregation Consortium, the ClinVar, the HGMD, the Infevers, and the 1000 Genomes Project. The prediction tools used for novel rare variants were SIFT, Polyphen 2, MutationTaster 2, CADD and UMD-Predictor. For each system, the most recent version would be used. Further, disease prevalence, expressivity, mode of inheritance and segregation would also be considered during data analyses. For all the disease-causing variants found by gene panel or WES, Sanger sequencing would be done for validation.

## Results

**Figure 2:**
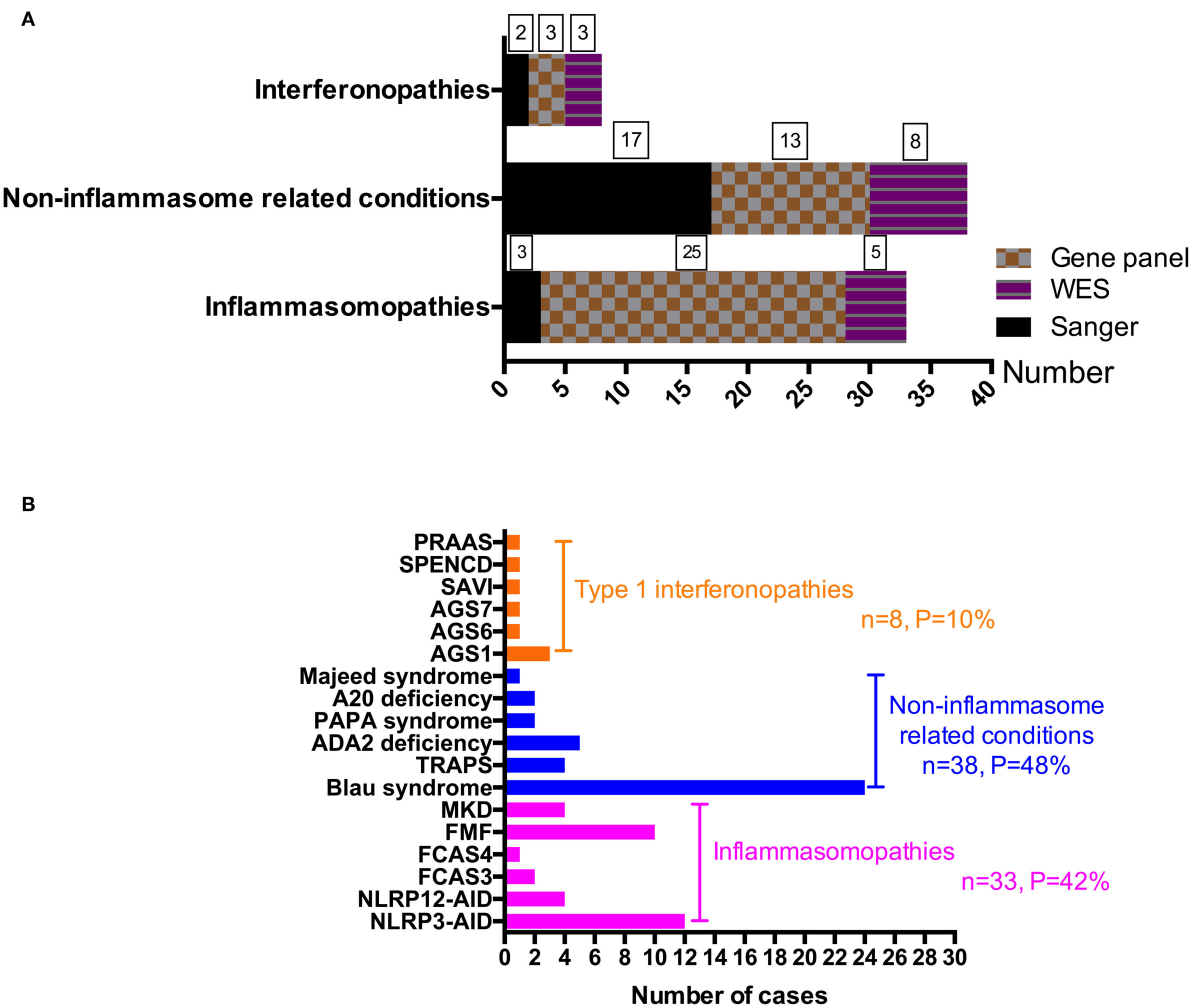
A summary of monogenic AIDs by disease group and disease itself. **(A)** A summary of gene detection methods used in different disease groups. **(B)** A presentation of each monogenic disorder diagnosed in our center.

From 2008 to 2019, 288 patients suspected of having monogenic AID went through genetic sequencing. As shown in Figures 1B, 2, we diagnosed 18 kinds of AID in 79 (27.4%) patients. Among them, 33 (42%) patients had inflammasomopathies, 38 (48%) had non-inflammasome related conditions, and 8 (10%) had type 1 interferonopathies. The genetic analysis methods used in each group of disorders are presented in Figure 2A. The clinical and genetic features of these three groups of patients are summarized below.

### Inflammasomopathies

We diagnosed 33 cases of inflammasomopathies with mutations in 6 genes, *NLRP3, NLRP12, NLRC4, PLCG2, MEFV*, and *MVK*. The detailed clinical and genetic information is listed in Table 1. For the 12 NLRP3 related autoinflammatory disease (NLRP3-AID) cases, which were previously known as cryopyrin-associated periodic syndromes (CAPS), the median age of onset was 0.2 months with a range of 0.1 months to 4.8 years, but the median age of diagnosis was 4.0 years with a range of 0.1 months to 20 years. The main clinical features were cutaneous rash, followed by fever, growth retardation, neurological abnormalities, lymphadenopathy, ocular manifestations, and arthralgia. All cases were autosomal dominant inherited and the variants were c.796C>T (p.L266F), c.913G>A (p.D305N), c.918G>T (p.E306D), c.932T>C (p.F311S), c.1049C>T (p.T350M), c.1311G>T (p.K437N), c.1711G>C (p.G571R), c.1715A>G (p.Y572C), c.1991T>C (p.M664T) and c.2113C>A (p.Q705K).

**Table 1:** Characteristics of Inflammasomopathies discovered in our center (Total *N* = 33).

**Characteristics (*N*, %)**	**NLRP3-AID (*n* = 12)**	**NLRP12-AID (*n* = 4)**	**FCAS3 (*n* = 2)**	**FCAS4 (*n* = 1)**	**FMF (*n* = 10)**	**MKD (*n* = 4)**
Male:Female	8:4	1:3	0:2	1:0	6:4	3:1
Age at onset (median, range)	0.2 m; (0.1 m−4.8 y)	4; (0–10)	12; (10–14)	1	7.5; (4–12)	0.6; (0.25–1)
Age at diagnosis (median, range)	4 (0.1 m−20 y)	11.5; (9–17)	15; (14–16)	2	10; (5–14)	2; (1–5)
Duration from onset to diagnosis (median, range)	4; (0–18)	7.5; (2–14)	3; (0–6)	1	1; (0–10)	1.5 (1–5)
Family history	0 (0)	0 (0)	2 (100)	0 (0)	0 (0)	1 (25)
Fever	11 (92)	4 (100)	2 (100)	1 (100)	10 (100)	4 (100)
Abdominal pain	0 (0)	1 (25)	0 (0)	0 (0)	5 (50)	1 (25)
Chest pain	0 (0)	0 (0)	0 (0)	0 (0)	0 (0)	0 (0)
Arthralgia/Arthritis	4 (33)	2 (50)	0 (0)	0 (0)	5 (50)	2 (50)
Myalgia	0 (0)	1 (25)	0 (0)	0 (0)	0 (0)	0 (0)
Cutaneous rash	12 (100)	3 (75)	2 (100)	1 (100)	5 (50)	3 (75)
Cold-induced urticarial	2 (17)	0 (0)	2 (100)	0 (0)	2 (20)	0 (0)
Facial edema	0 (0)	0 (0)	1 (50)	1 (100)	0 (0)	0 (0)
Neurological abnormalities	7 (58)	0 (0)	0 (0)	0 (0)	0 (0)	0 (0)
Oral ulcer	0 (0)	0 (0)	1 (50)	1 (100)	1 (10)	1 (25)
Ocular manifestations	5 (42)	1 (25)	0 (0)	0 (0)	0 (0)	0 (0)
Lymphadenopathy	6 (50)	1 (25)	2 (100)	0 (0)	1 (10)	2 (50)
Hepatosplenomegaly	2 (17)	1 (25)	1 (50)	0 (0)	1 (10)	1 (25)
Otological abnormalities	5 (42)	2 (50)	0 (0)	0 (0)	0 (0)	0 (0)
Vasculitis	0 (0)	0 (0)	0 (0)	0 (0)	1 (10)	0 (0)
Amyloidosis	0 (0)	0 (0)	0 (0)	0 (0)	0 (0)	0 (0)
Growth retardation	9 (75)	0 (0)	0 (0)	0 (0)	1 (10)	2 (50)
ANA positivity	2 (17)	1 (25)	1 (50)	0 (0)	1 (10)	0 (0)
Elevated ESR/CRP	12 (100)	4 (100)	2 (100)	1 (100)	10 (100)	4 (100)
Genes	*NLRP3*	*NLRP12*	*PLCG2*	*NLRC4*	*MEFV*	*MVK*
Inheritance	AD	AD	AD	AD	AR	AR
Gene mutations/variants	p.L266F p.D305N p.E306D p.F311S p.T350M p.K437N p.G571R p.Y572C p.M664T p.Q705K	p.L558R p.W581X p.G730R c.2072+2 dupT	p.C1082R p.I1175K	p.G172S	p.L110P p.P115R p.E148Q p.E230K p.G304R p.P369S p.P633L p.F636Y	p.V8M/p.G336S p.S118P/p.G211X p.G219R/p.P263L

*AD, autosomal dominant; AR, autosomal recessive; FCAS, familial cold autoinflammatory syndrome; FMF, familial Mediterranean fever; MKD, mevalonate kinase deficiency; m, month; y, year*.

For the 4 NLRP12-AID cases, the median age at disease onset was 4.0 years. Fever, skin rash, arthritis and ontological disorders were the most prevalent features. They each carried a heterozygous germline mutation including c.1673T>G (p.L558R), c.1742G>A (p.W581X), c.2188G>C (p.G730R) and c.2072+2dupT accordingly, which have been analyzed and reported previously (12).

We also diagnosed two familial cold autoinflammatory syndrome 3 (FCAS3) caused by *PLCG2* gain-of-function mutations. Both patients had fever, cold-induced urticarial rash and lymphadenopathy. One also developed facial edema and oral ulcer while the other one had hepatosplenomegaly. Both patients had family history with dominantly inherited traits. Though they had significantly elevated CRP and ESR, neither had antibody deficiency nor recurrent infections, which are characteristics of APLAID (*PLCG2*-associated antibody deficiency and immune dysregulation) syndrome (13). The two mutations were c.3244T>C (p.C1082R) and c.3524T>A (p.I1175K). These were two novel mutations.

NLRC4 inflammasomopathy has varied phenotypes including autoinflammation and infantile enterocolitis (AIFEC) and familial cold autoinflammatory syndrome 4 (FCAS4) (14). As for our patient, he carried a novel mutation, c.514G>A (p.G172S), in nucleotide binding domain (NBD) of NLRC4. He had urticarial-like skin rashes since 3 months of age. Later he had periodic fever once every 3–6 months. Like S171F, this mutation would substitute a hydrophilic serine residue for a hydrophobic glycine residue at the adenosine diphosphate (ADP)-binding interface within the conserved NBD region, which disrupts the auto-inhibitory mechanism maintaining the inactive state of NLRC4 (15).

We also discovered 10 FMF patients over the past decade. The median age at disease onset was 7.5 (range, 4–12) years, while the median age at diagnosis was 10 (range, 5–14) years. Apparently, fever was the most common symptom, followed by abdominal pain, arthralgia and rash. Other less prevalent symptoms included oral ulcer, vasculitis, lymphadenopathy, hepatosplenomegaly and growth retardation. No amyloidosis was found in these patients. The *MEFV* variants of these patients were c.329T>C (p.L110P), c.344C>G (p.P115R), c.442G>C (p.E148Q), c.688G>A (p.E230K), c.910G>A (p.G304R), c.1105C>T (p.P369S), c.1898C>T (p.P633L), c.1907T>A (p.F636Y), and c.1759+8C>T. Each patient carried 2–5 variants, and E148Q was the most common one. Six patients responded well to colchicine treatment.

The last disease in this section is mevalonate kinase deficiency (MKD), which is an autosomal recessive disorder caused by mevalonate kinase gene (*MVK*) mutations. Defects in the mevalonate kinase pathway can promote pyrin inflammasome activation by dysregulating geranylgeranylation and function of small cellular GTPases (16). We discovered four patients in total and they all developed symptoms soon after birth. The median age at diagnosis was 2.0 years (range, 1–5) and the median delay of diagnosis was 1.5 years (range, 1–5). All of them had typical periodic fever, while other symptoms were cutaneous rash, arthralgia/arthritis, growth retardation, gastrointestinal problems, and lymphadenopathy (25%). The genetic mutations were p.V8M, p.S118P, p.G211X, p.G219R, p.P263L, and p.G336S.

### Non-inflammasome Related Conditions

We diagnosed 38 cases under this branch, and a brief summary is listed in Table 2.

**Table 2:** Characteristics of the non-inflammasome related conditions.

**Disease**	**Age onset** **(median, range)**	**Gender** **(male: female)**	**Main clinical findings**	**Genetic defects**	**Inheritance**
Blau syndrome (*n* = 24)	1 (0–4)	11:13	Arthropathy (19/24), rash (13/24), uveitis (7/24), growth retardation (7/24), camptodactyly (6/24)	*NOD2* p.R334Q, p.R334W, p.E383Q, p.G481D, p.M491L, p.E498G, p.D512Y, p.M513T, p.R587C, p.H603D, p.H669R	AD
TRAPS (*n* = 4)	0.5 (0–1)	4:0	Periodic ever (4/4), rash (3/4), arthritis (3/4), abdominal pain (2/4) lymphadenopathy, (2/4)	*TNFRSF1A* p.C99S p.T79M p.F141C	AD
ADA2 deficiency (*n* = 5)	3.2 (0–9)	3:2	Fever, rash, polyarteritis nodosa, early-onset recurrent stroke	*ADA2* p.N85I / p.G284V p.H293P / p.Y88C p.G5R / exon 7 deletion, p.R169Q / p.R131Sfs*52 p.Y411C / p. N328I	AR
PAID (*n* = 2)	2 (0–4)	1:1	Fever, hematological problems, arthritis, rash	*PSTPIP1* p.E250K, p.N236K	AD
A20 deficiency (*n* = 2)	4 (0–8)	0:2	Fever, rash, arthralgia, mucosal ulcers, bowel inflammation, CTD	*TNFAIP3* p.R45X, p.R271X	AD
Majeed syndrome (*n* = 1)	0.2	1:0	Fever, osteomyelitis, congenital anemia, skin disorders	*LPIN2* c.2327+1G>C c.1691_1694delGAGA	AR

*AD, autosomal dominant; AR, autosomal recessive; CTD, connective tissue disease*.

Blau syndrome was initially described in 1985 with a triad of granulomatous arthropathy, uveitis, and dermatitis (17). Later in 2001, *NOD2* was discovered to be the causative gene, defects of which promote hyperactivation of NF-κB signaling and inflammation processes. A total of 24 patients were genetically diagnosed and all of them developed symptoms before 5 years old. Overall, 79% cases had granulomatous arthropathy, 54% cases had scaly rash/dermatitis, and 29% cases had bilateral uveitis. The uveitis was relatively severe because three cases turned out to be near blind regardless of treatment. Unfortunately, 13 patients were misdiagnosed as other diseases such as juvenile idiopathic arthritis (JIA) and atopic dermatitis before referral to our center. As for the *NOD2* mutations, all of them were located within or near the NOD/NACHT domain of the NOD2 protein, with p.R334Q (7/24, 29%) and p.R334W (7/24, 29%) being the most common ones. Other mutations were p.E383Q, p.G481D, p.M491L, p.E498G, p.D512Y, p.M513T, p.R587C (2/24, 8.3%), p.H603D, and p.H669R. Of these mutations, p.M491L, p.E498G, and p.H669R were novel variants.

Another classical non-inflammasome related condition is the TNF receptor-associated autoinflammatory syndrome (TRAPS), a dominantly inherited disorder caused by mutations in the TNF receptor (TNFR1) encoded by the TNF superfamily receptor 1A (*TNFRSF1A*) gene (18). Fever is often long lasting and can be accompanied by arthritis, a patchy migratory skin rash, serositis, and periorbital edema. All the four patients developed severe symptoms under 1 year of age. All of them had prolonged periodic fever, while both rash and arthritis occurred in three patients each. Two patients had abdominal pain, a sign of serositis, and two patients had overt reactive lymphadenopathy. The mutations of TNFRSF1A were p.C99S (2/4, 50%), p.T79M, and p.F141C, which were not reported elsewhere. Since TNFR1 is a transmembrane glycoprotein containing four tandem-repeat cysteine-rich domains (CRD1-4) (19), introducing (p.F141C) or removing (p.C99S) cysteine residues were believed to affect molecular structures and cause more severe phenotypes. The p.T79M mutation might disrupt hydrogen bonds between proteins.

Deficiency of adenosine deaminase type 2 (DADA2) is an autosomal recessive monogenic AID caused by *ADA2* (formerly known as *CECR1*) mutations and was first described in 2014 in patients with mild immune deficiency, systemic inflammation, and central nervous system vasculopathy (20). ADA2 deficiency leads to accumulation of adenosine, which then activates neutrophils and dysregulates macrophage differentiation to cause inflammation, damage and fibrosis of many tissues (21). We diagnosed five patients and the median age of onset was 3.2 years old. The main clinical features of these patients were fever (100%), rash (100%), livedo racemosa (40%), and early-onset recurrent stroke (20%). The genotypes for these patients were p.N85I/p.G284V, p.H293P/p.Y88C, p.G5R/p.V325Tfs^*^7 (exon 7 deletion), p.R169Q/p.R131Sfs^*^52, and p.Y411C/p. N328I accordingly. All these variants have not been reported other than p.V325Tfs^*^7, p.R169Q and p.N328I.

We also diagnosed two patients of *PSTPIP1*-associated inflammatory diseases (PAID), a dominantly inherited disease that can be subtyped into pyogenic arthritis, pyoderma gangrenosum, and acne (PAPA) syndrome, *PSTPIP1*-associated myeloid-related proteinemia inflammatory (PAMI) syndrome, and other PAPA-like syndromes (22). Both patients had skin rash, arthritis, persistent systemic inflammation, hepatosplenomegaly, pancytopenia, and growth retardation, which matched the typical phenotypes of PAMI. The genotypes were *de novo* mutations of p.E250K and p.N236K, resulting in charge changes of the F-BAR domain of PSTPIP1 and increased binding to pyrin (23).

We also diagnosed two cases of haploinsufficiency of A20 (HA20). A reduced expression of A20, encoded by *TNFAIP3*, leads to insufficient suppression of NF-κB activity and enhanced NLRP3 inflammasome activation, thereby generating large amounts of pro-inflammatory cytokines (24). The clinical phenotypes of the disorder are quite variable. For our patients, both suffered from recurrent oral ulcer. One patient was diagnosed as Behçet disease previously and the other one was diagnosed with undifferentiated connective tissue disease. Hashimoto′s thyroiditis was also found in one patient. The genetic sequencing yielded p.R271X of the *TNFAIP3* gene in one patient and p.R45X in the other one, which were known to be pathogenic variants (25, 26).

The last disease in this section is Majeed syndrome, which is an autosomal recessive disorder due to mutations in *LPIN2* that encodes the protein LIPIN2, a negative regulator of NLRP3 inflammasome (27). Early reports documented a classic triad of clinical findings including severe early onset chronic osteomyelitis, microcytic congenital dyserythropoietic anemia, and the neutrophilic dermatosis (28). Our case was a male patient who developed fever and bone pain soon after birth, and later presented with dermatosis. Diagnostic workup revealed highly elevated acute phase reactants, microcytic anemia and chronic osteomyelitis. Whole exome sequencing revealed c.2327+1G>C and c.1691_1694delGAGA of *LPIN2*. The c.2327+1G>C is a reported pathogenic mutation locating at a splice site of intron 17 and the c.1691_1694delGAGA is a frameshift mutation that has not been reported elsewhere.

### Type 1 Interferonopathies

In total, we diagnosed five cases of AGS, one case of stimulator of interferon genes (STING)-associated vasculopathy with onset in infancy (SAVI), one case of spondyloenchondrodysplasia with immune dysregulation (SPENCD), and one case of PRAAS/CANDLE. The clinical characteristics and genotype information are summarized in Table 3. For AGS1 that caused by *TREX1* mutations, rash, autoimmune features and intracranial calcifications were the main phenotypes. All of the variants were not reported previously. For AGS6, a patient with c.305_306del (p.Q102Rfs^*^22) of *ADAR1*, pulmonary artery hypertension (PAH) was noted in addition to chilblain rash, lupus phenotype and intracranial calcification. As for the case of AGS7, the age of disease onset was 0.5 years. He had prominent growth retardation, cutaneous rash and severe leukopenia. His interferon signature of the peripheral blood was high and the genetic analysis yielded p.A339D *de novo* mutation of *IFIH1*. He received Janus kinase (JAK) inhibitor treatment after diagnosis and remains stable till now. We also diagnosed 1 SAVI patient, who had severe PAH and interstitial lung disease at the time of first visit to our center. His lungs were already fibrotic but did not get worse after treatment with JAK inhibitors. As for the case of SPENCD, her initial chief of complaint was intermittent fever. During follow up period, she started to present features of systemic lupus erythematosus and short stature. Her spine X-ray revealed spondyloenchondrodysplasia. This is an autosomal recessive inherited disorder and the genetic variants of this patient were p.S267Lfs^*^20 and p.G239D of *ACP5*. For the PRAAS/CANDLE patient, the most prominent characteristic was lypodystrophy, other clinical features included fever, rash, PAH, arthritis, intracranial calcification, uveitis, loss of hearing, and growth retardation. He had compound heterozygous mutations of *PSMB8*, which were p.T75M and p.T74I.

**Table 3:** Characteristics of the eight cases diagnosed as interferonopathies.

**Case**	**Clinical presentation**	**Age onset**	**Genetics**	**Diagnosis**	**Inheritance**	**Intervention**
1	Rash, growth retardation, intracranial calcification	1y	*TREX1*, c.505C>T, p.R169C; c.900delA, p.S301Lfs*31	AGS1	AR	Prednisone DMARD
2	Rash, glaucoma, intracranial calcification, lupus	3y	*TREX1*, c.139G>A, p.G47S c.458dupA p.C154Mfs*3	AGS1	AR	JAK inhibitor
3	Rash, mixed connective tissue disease, ILD	3y	*TREX1*, c.65C>T, p.T22M	AGS1	AD	JAK inhibitor
4	Lupus, rash, arthritis, PAH, intracranial calcification	3y	*ADAR1*, c.305_306del, p.Q102Rfs*22	AGS6	AD	Prednisone DMARD
5	Growth retardation, rash, intracranial calcification, leukopenia	6m	*IFIH1*, c.1016C>A, p.A339D	AGS7	AD	JAK inhibitor
6	Rash, growth retardation, PAH, ILD	1m	*TMEM173* c.463G>A, p.V155M	SAVI	AD	JAK inhibitor
7	Fever, bone dysplasia, nervous system problems, autoimmune disease	2y	*ACP5* c.798duC, p.S267Lfs*20; c.716G>A, p.G239D	SPENCD	AR	JAK inhibitor
8	Fever, rash, PAH, lipodystrophy, arthropathy, intracranial calcification, growth retardation	7m	*PSMB8* c.224C>T, p.T75M; c.221C>T, p.T74I	PRAAS	AR	Death prior to treatment

*AD, autosomal dominant; AGS, Aicardi Goutieres syndrome; AR, autosomal recessive; ILD, interstitial lung disease; PAH, pulmonary artery hypertention; PRAAS, proteasome-associated autoinflammatory syndrome; SAVI, STING associated vasculopathy with onset in infancy; SPENCD, spondyloenchondrodysplasia with immune dysregulation*.

## Discussion

Human innate immunity is first-line defense against the microbial world, and also serves as a guardian to limit danger molecules and prevent self-invasion. While an inadequate innate immune response can lead to severe infections, persistent excessive responses can generate systemic autoinflammatory disorders, most of which are monogenic inborn errors. Although just conceptualized around 20 years ago, the field of AIDs sitting within the domains of rheumatology and clinical immunology has expanded ever since. Here, we report the first and also the largest cohort of monogenic AIDs in Chinese pediatric population diagnosed over the past decade.

**Figure 3:**
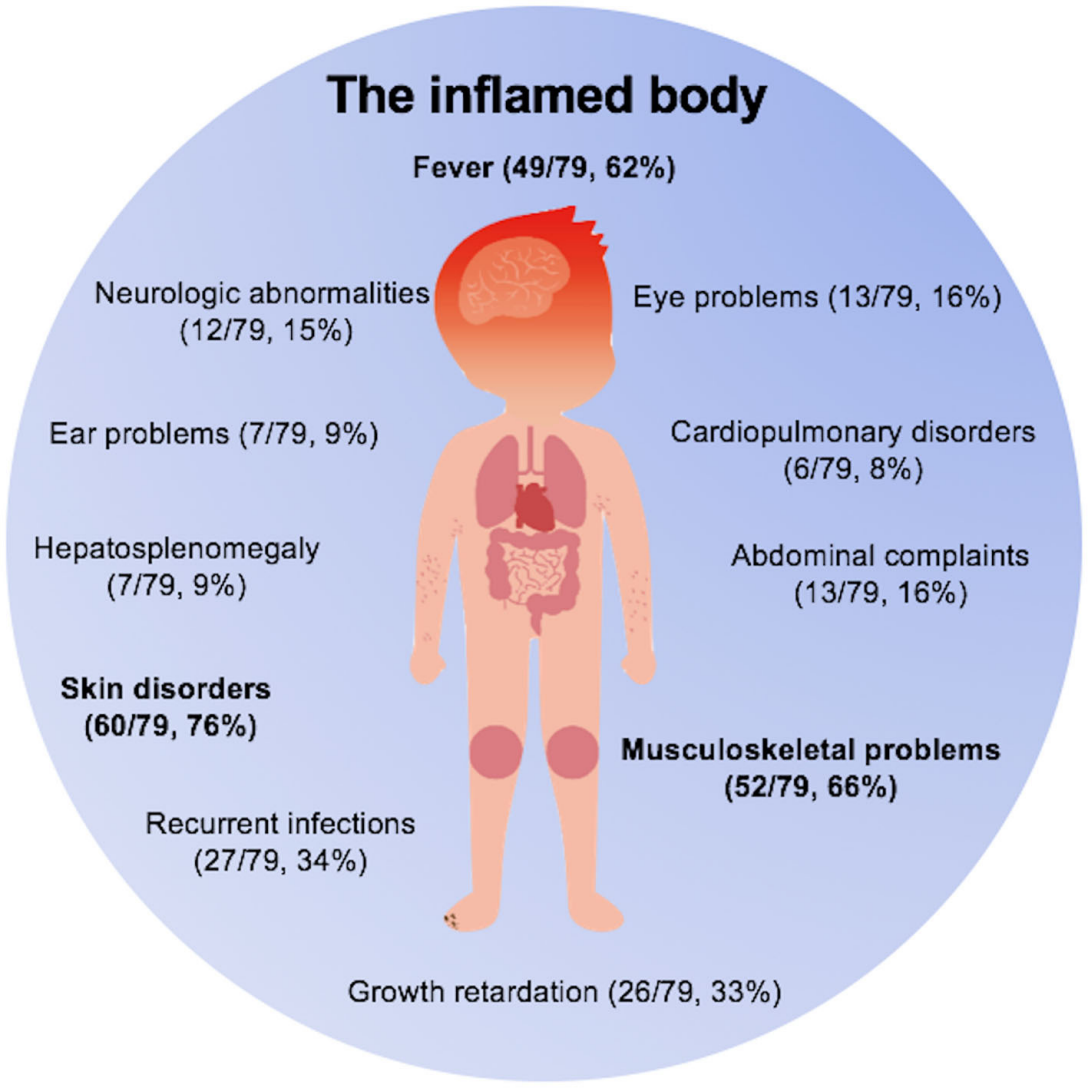
The inflamed body cartoon. Fever, skin disorders, and musculoskeletal problems were three main clinical features of monogenic AIDs.

Overall, in our 79 monogenic AID patients, as depicted in Figure 3, skin disorders (76%), musculoskeletal problems (66%), and fever (62%) were the most common clinical features. Other features included central nervous system abnormalities (15%), eye disorders (16%), ear problems (9%), cardiopulmonary disorders (8%), and symptoms involving gastrointestinal tract system (25%). In addition to above presentations, one third of the sick kids would experience episodes of infections because of the dysregulated innate immunity. We also noticed that growth retardation was relatively common among these children (33%). Among those 26 patients with growth retardation, a total of 11 patients had used glucocorticoids (GC). Only four of the 11 patients had received GC treatment for a long time and they were all Blau syndrome patients. Therefore, growth retardation is a kind of phenomenon worthy of attention in children with prolonged uncontrolled inflammation. We also need to address that there were 24 patients (30%) in total misdiagnosed as other diseases, with juvenile idiopathic arthritis being the most prevalent one (12/79), which prompts us pediatric rheumatologists to consider monogenic AIDs when making diagnosis of JIA.

Arriving at an accurate diagnosis of monogenic AID is quite difficult if only through reading clinical phenotypes. The genomic analysis has been integrated into the whole diagnosis process over the past 20 years and seen many breakthrough discoveries. Our center started screening AID genes in highly suspected AID patients such as patients with periodic fever in the year of 2008 with Sanger sequencing. Later we integrated primary immunodeficiency (PID) gene panel in 2012, which helped us diagnose more than half of these patients (46/79, 58%) and are still in current use. For untypical cases with prominent inflammatory phenotypes or typical cases with negative panel screening results, we used WES method, which dated back to the year of 2015. The WES method discovered 16 AID patients (16/79, 20%) in total and is playing a very important role in discovering and characterizing new disorders. Overall, the positive rate of genetic sequencing in our center was 27.4% (79/288). There might be several reasons for this. First, sequencing approach such as whole exome sequencing might miss deep intronic variants that can affect protein translation and expression. Second, disorders like periodic fever syndromes can be affected by environmental triggers through inducing epigenetic changes of specific genes, which cannot be detected by normal genetic sequencing methods (29). Third, a highly suspected AID with negative genetic test results could simply be due to misinterpretation of the genetic results or unknown genes to be identified. As such, the clinical diagnosis can't be excluded by the negative results of genetic sequencing and a further periodic re-evaluation and re-analysis of the genetic variants is highly recommended for undefined AID patients. We have to admit that in real-life clinical setting, there are indeed a small number of patients with typical phenotypes of periodic fever syndromes and negative genetic test results can be clinically diagnosed. However, these seemingly typical cases were not included in this study, because we observed the occurrence of malignancy in several cases during the long-term follow-up period. For suspected AID patients with unusual phenotypes, the interpretation of variant pathogenicity should be cautious and performed repeatedly under appropriate standardized protocol, especially for the genes that do not seem relevant to the phenotype of the patient.

The high-throughput sequencing technologies not only boost the pace of discovery of new monogenic AIDs, but also further expand our understanding of the innate immunity and help develop targeted therapies to treat autoinflammation. Since the autoinflammation largely depends on cytokines, cytokine directed therapies such as IL-1 inhibition, anti-TNF-α agents and JAK inhibitors have been used in monogenic AID patients (30). However, IL-1 inhibitors are not available in Mainland of China, most of our NLRP3-AID patients were treated by steroids or IL-6 inhibition with low to moderate efficacy. For Blau syndrome, DADA2 and HA20 patients, we initiated anti-TNF-α therapies and have observed some good responses. For type 1 interferonopathies, we tested JAK inhibitors including ruxolitinib and tofacitinib among these patients and the results were promising. All of these AID patients are under regular follow-up and the data pertaining to treatment plans and efficacy would be revealed in later reports.

This report is important because most of the monogenic AID studies were performed in Western countries and the data is scarce in Chinese pediatric population. The clinical characteristics and genetic information provided by our center can help increase the awareness of monogenic AIDs and further avoid misdiagnoses, unnecessary hospitalizations, and inappropriate therapeutic treatment. However, there are some limitations of this study. First, for undefined AID patients who initially received Sanger sequencing of the highly suspected genes, the sensitivity would be lower than in reality, even though we have tried to perform re-analysis in most of those patients. Second, the total number of patients of this report is still limited, and the information provided here is only a reflection of monogenic AIDs diagnosed in a tertiary pediatric center, although our center is already one of the biggest pediatric rheumatology centers in mainland of China. We aim to set up a national network and collaborate with other centers in the near future to collect more solid data and perform more detailed analyses such as phenotype-genotype correlation in each disease in Chinese pediatric population. Third, for the newly discovered variants with unknown pathogenicity, we only used prediction software and did not validate them experimentally. However, we had rounds of discussions about those variants and correlating phenotypes, and had followed up those patients with undefined variants at a regular basis for better disease characterization.

## Conclusions

In conclusion, the present study described the first cohort of monogenic AID patients in Chinese pediatric population. A total of 79 monogenic AID patients were diagnosed, with fever, skin problems, and musculoskeletal system disorders being the most prevalent clinical features. We totally found 18 kinds of monogenic AIDs with the help of genetic sequencing, and many of the variants in this cohort were newly discovered. By providing data from our center, we hope this report would reflect and also expand the phenotypic and genotypic profiles of Chinese pediatric patients with monogenic AIDs.

## Data Availability Statement

The original contributions presented in the study are included in the article/supplementary material, further inquiries can be directed to the corresponding author/s.

## Ethics Statement

The studies involving human participants were reviewed and approved by Institutional Review Board of the Peking Union Medical College Hospital. Written informed consent to participate in this study was provided by the participants' legal guardian/next of kin.

## Author Contributions

WW and ZY contributed to the conceptualization, methodology, and writing of the original draft. WW performed genetic sequencing and genetic data analysis. ZY, LG, LZ, JL, MM, CW, YZ, YR, ZS, QW, YD, and HS managed patients and collected data. HS contributed to conceptualization, methodology, writing of the original draft, editing, funding acquisition, and supervision. All authors approved the final version of the manuscript.

## Conflict of Interest

The authors declare that the research was conducted in the absence of any commercial or financial relationships that could be construed as a potential conflict of interest.
